# The Potential of Clinical Decision Support Systems for Prevention, Diagnosis, and Monitoring of Allergic Diseases

**DOI:** 10.3389/fimmu.2020.02116

**Published:** 2020-09-10

**Authors:** Stephanie Dramburg, María Marchante Fernández, Ekaterina Potapova, Paolo Maria Matricardi

**Affiliations:** Department of Pediatric Pulmonology, Immunology and Critical Care Medicine, Charité-Universitätsmedizin Berlin, Berlin, Germany

**Keywords:** CDSS, digital health, allergy, clinical decision support systems, prevention

## Abstract

Clinical decision support systems (CDSS) aid health care professionals (HCP) in evaluating large sets of information and taking informed decisions during their clinical routine. CDSS are becoming particularly important in the perspective of precision medicine, when HCP need to consider growing amounts of data to create precise patient profiles for personalized diagnosis, treatment and outcome monitoring. In allergy care, several CDSS are being developed and investigated, mainly for respiratory allergic diseases. Although the proposed solutions address different stakeholders, the majority aims at facilitating evidence-based and shared decision-making, incorporating guidelines, and real-time clinical data. We offer here an overview on existing tools, new developments and novel concepts and discuss the potential of digital CDSS in improving prevention, diagnosis and monitoring of allergic diseases.

## The Evolution of Clinical Decision Support Systems in the Context of Precision Medicine

### Origins of Digital Clinical Decision Support

Researchers and engineering technologists have been developing and exploring computerized decision support systems (DSS) for approximately 50 years (1). In their early stages, DSS were categorized according to their methodology, ranging from data-oriented approaches, whose systems merely extracted information, to model-oriented concepts, mostly focused on decision processes. Alongside technological advances, this kind of computerized tool became increasingly powerful and elaborated, offering solutions for the processing of complex data sets and their integration in decision algorithms providing data-driven suggestions to the user (2). The ability to capture expert knowledge, guidelines and reasoning techniques, together with the automation of rules via identification of key attributes led to the development of new digital support opportunities for healthcare providers in their clinical routine (3).

### Methodologies

The development of computerized clinical support tools can be grouped according to the following main methodologies: (a) information retrieval tools to answer clinical questions and manage medical information (4); (b) logical models for the assignment of categories for medical standard measurements (5), characterized alerts and reminder systems (6); (c) probabilistic and data-driven prediction algorithms to improve patient outcomes (7); and (d) a modeled combination of formal and heuristic algorithms supporting physicians in their decision on the individual deployment of evidence-based solutions (8, 9). Today, these methodologies carry the potential of becoming an innovative resource for digital augmentation of clinical care, once scientifically and clinically validated. To ensure patient safety, technologies with a potential impact on medical decisions need to undergo the registration and certification process for medical devices at the respective regulatory authorities (10, 11).

### Advantages of Digital Decision Support

Due to the growing amount of health data, delivering personalized precision medicine has become a challenging task (12). Large sets of information are not only derived from complex diagnostic test systems, genetic analyses and –omics approaches, but also patient-generated monitoring data, exposure information and/or the surveillance of physiological parameters via smart devices and sensors (13). In parallel, electronic health records (EHR) have become a common platform to bundle patient data and clinical decision support systems (CDSS) are frequently connected to these critical information hubs (6). This is where trained algorithms and validated computerized tools can assist health care professionals (HCP) in efficiently interpreting complex data sets and keeping the overview on the individual health status of a patient (14). Automated monitoring and alerts systems further enable a continuous personalized treatment follow-up and allow an early intervention in the case of side effects or insufficient success.

### Challenges and Limitations of Clinical Decision Support

Although digital decision support tools can be potentially useful in optimizing clinical workflows, it is important to mention that they are by no means able to replace a trained healthcare professional. In addition, several challenges need to be considered. Clinical work often includes hectic situations and a broad spectrum of patients with different conditions, comorbidities, and treatment plans being treated by one doctor. Therefore, a support tool needs to be extraordinarily user-friendly, ideally adaptable to individual settings with a high level of interoperability and include a solid risk management as any medical device. Apart from technical challenges like an optimal human-computer interface there are particular aspects to be addressed within different methodologies (1). To give an example: alert systems with an high alert frequency (e.g., for potential drug interactions) may cause a alert fatigue in the user, who may decide more frequently to disregard the suggestions. As the user is a trained professional, this may not seem crucial at first sight. However, a negative impression of fatigue may lead to an adverse predisposition toward other, potentially more user-friendly, technologies. This example reflects the diversity of challenges, which need to be considered in the development of decision support tools. They are not only of technical nature but also related to adoption by different stakeholders and integration into a broad spectrum of pre-existing settings.

### Adoption of Decision Support Technologies by Health Care Professionals

**FIGURE 1:**
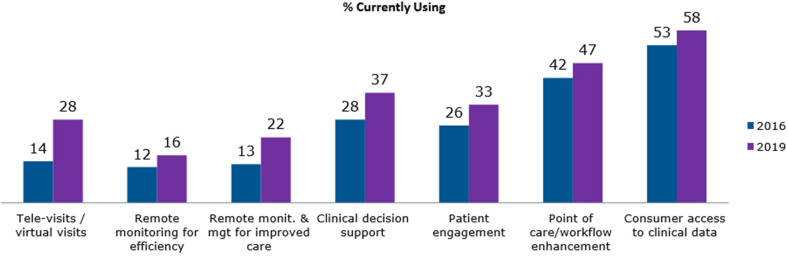
The use of digital health tools among United States physicians in 2016 and 2019. The survey has been performed by the American Medical Association (AMA) among 1300 (1359 respectively) physicians working in different clinical settings. © 2020 American Medical Association. Reprinted with Permission [17]. https://www.ama-assn.org/.

The prospective collection of clinical and diagnostic data provides valuable insights into disease endo- and phenotypes and has the potential to offer distinct advantages in the field of chronic diseases such as allergy and asthma (15). Digital technologies potentially allow continuous disease monitoring with agile adaptation strategies decided on by the physician to improve patient outcomes and quality of life, especially as part of a chronic disease management process. However, physicians are core to the medical decision process and accountable for their choices. If computer algorithms are aiding these, reliability and accountability are key elements to be addressed prior to a widespread adoption of any digital solution. This challenge may be one of the reasons for low adoption rates for new digital tools in clinical routine (16), although recent data from the American Medical Association show an increased interest among physicians in digital support tools (from 28% in 2016 to 37% in 2019) (17). The category CDSS in this assessment included any modules and integrated mobile applications in conjunction with EHR, also enabling the remote monitoring of patient-related parameters and automated integration of the results in the central data set. Interestingly, the adoption rates for monitoring tools alone, without integration in a CDSS, increased from 12% (2016) to 16% (2019) for remote efficiency monitoring and from 13% (2016) to 22% (2019) for remote management tools for chronically ill patients (17) (Figure 1). It has to be stated, though, that reported use does not necessarily reflect any improvement of patient outcomes (18). In order to enable a critical evaluation and smooth implementation of new tools in healthcare systems, it is important to ensure that professionals are adequately trained on the benefits and challenges of CDSS before applying them in clinical practice (9).

## Clinical Decision Support for Allergic Diseases

Allergic diseases are clinically and immunologically multifaceted, as well as pathogenetically heterogenous (15), which makes an evidence-based clinical diagnosis rather challenging. Moreover, the ratio of allergists per allergic patient is in general rather low and highly heterogenous in different countries (19, 20). As a first point of contact, most allergic patients see a primary care doctor, who frequently lacks the knowledge, confidence or resources to meet their specific needs due to insufficient training (21). Independently from the individual level of specific training, clinicians are confronted with a broad spectrum of clinical manifestations, such as allergic rhinitis (AR), asthma, atopic eczema, food allergies, anaphylaxis, drug allergies or occupational allergies, whose pathogenesis is heterogeneous. In addition, the nomenclature and classification of these allergic diseases is being challenged (22, 23) and the guidelines for diagnostic work-ups change over time (24, 25) or differ between different regions (26, 27).

In this context, computerized decision support concepts are becoming a potentially valuable tool to support clinicians in evaluating large data sets and taking into account complex guidelines. Electronic health (eHealth) technologies, especially mobile health (mHealth) tools, have become more and more popular and provide valuable clinical information on patients. However, tools merely providing information without concrete suggestions for diagnostic and therapeutic decisions should not be considered a CDSS. The software tools and mobile solutions discussed here have the potential to enhance medical decisions at the point-of-care mainly with (A) targeted patient information, (B) guideline- and evidence-based clinical knowledge, and (C) prospectively collected data (patient-/sensor-generated). A positive effect of clinical decision support on the practitioner’s performance has already been shown for several chronic health conditions (28–31). However, more studies are needed to assess the impact on short, medium-, and long-term patient outcomes. In allergy care, several concepts for CDSS have been created, addressing different diseases and settings with the aim of improving detection, diagnosis and diseases management (32, 33). In most cases, the main target is to empower not only the allergist, but also the general practitioners (GPs) and even the patient, at public contact points such as pharmacies. The following paragraphs will give an overview on existing solutions, concepts and potentials for future developments.

## Decision Support Systems for the Management of Allergic Rhinitis

### Developments of the Allergy and Its Impact on Asthma (ARIA) Consortium

Several expert groups have elaborated algorithms and support tools to facilitate screening, diagnostic precision, early optimization of therapy and user-friendly monitoring of chronic respiratory allergic diseases. Among these, the ARIA consortium elaborated a detailed decision algorithm based on clinical scenarios for AR patients treated with symptomatic drugs. The development process involved a key opinion leader consensus on specific treatment recommendations, which has been published transparently (34). The authors count on a broad experience on the collection of symptom data via mobile health technology (35–37) in which the CDSS is planned to be integrated. However, the system is not yet publicly available online and publications on its implementation are expected to be published soon.

### DSS in the Pharmacy

**FIGURE 2:**
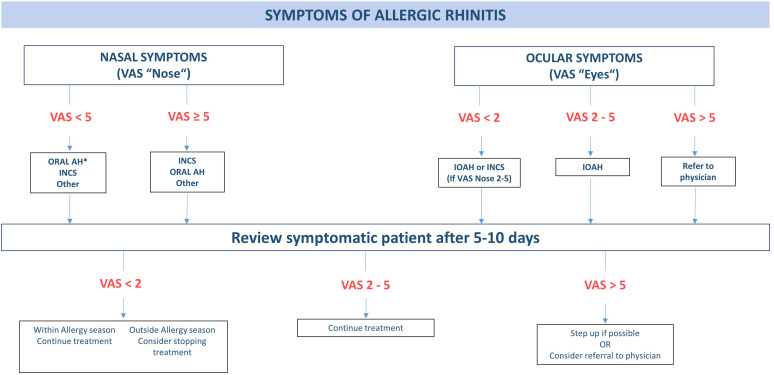
Decision algorithm for treatment of allergic rhinitis in the pharmacy. AH, antihistamine; INAH, intranasal antihistamine; INCS, intranasal corticosteroid; IOAH, intraocular antihistamine. *INCS if coexisting asthma. Visual Analogue Scale (VAS) nose/eye: “How much are your nose/eye symptoms bothering you today?” (0 = not at all bothersome, 10 = extremely bothersome). Adapted from Tan et al. (38).

Another ARIA initiative to provide front-line decision support has been proposed to implement integrated care pathways for AR at the level of community pharmacies (37). Patients suffering from AR often self-medicate with over-the-counter-drugs with correspondingly poor results (38); hence, pharmacists assume an important role in the care pathway for patients suffering from respiratory allergies (39). An open intervention study among German pharmacists revealed that pharmacists failed to ask several questions essential to make a diagnosis, confirm the appropriateness of self-medication and the drug choice (40). When implementing a pharmacist decision support system (PDSS), the pharmacists asked seven (78%; IQR 5.25–9) instead of two (22%; IQR 1–3) of the nine required questions. The use of the PDSS resulted in a significant improvement of patient evaluation and required only 1.5 min (40). Notwithstanding its limitations, this study pioneers the implementation of a DSS for AR symptomatic treatment at pharmacy level. The ARIA consortium also underlined the importance of pharmacists for integrated care pathways of respiratory allergic diseases (41). The authors recently proposed a CDSS supporting pharmacists in monitoring the patient’s symptom control and adjusting symptomatic treatment accordingly (42) (Figure 2). This comprehensive approach to supporting pharmacists in their front-line role in allergy care is a promising concept. The evaluation or validation of the system in a real-life setting is therefore a research priority.

### CDSS for Allergen Immunotherapy

**FIGURE 3:**
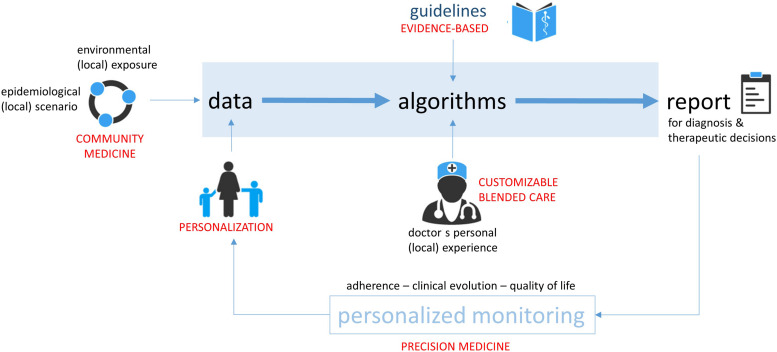
General concept of a guideline-based, looped and customizable clinical decision support system. Thresholds can be adapted by the clinician according to his/her clinical experience and local characteristics in a blended care setting. Patient and environmental data are continuously collected, and updated reports created. Adapted from Matricardi et al. (33).

A different concept has been followed in the development of a support system facilitating the precise prescription of allergen-specific immunotherapy (AIT), i.e., the only disease-modifying treatment for AR (33). Decision support for AIT prescription is particularly valuable in geographic areas with high rates of poly-sensitization and overlapping flowering periods of many allergenic plants; in these areas the distinction between genuine and cross-reactive sensitization is extremely difficult and the precise identification of the pollen causing allergic symptoms is crucial for AIT efficacy (43, 44). The traditional diagnostic approach, based on a retrospective clinical history and extract-based skin prick or IgE testing, is suboptimal due to several reasons. On one hand, the clinical history can be unreliable due to a recall bias, especially when seasonal symptoms occurred several months before the patient’s interview. On the other hand, extract-based diagnostic tests are unable to distinguish a genuine sensitization from cross-reactivity due to poor standardization and high sequence homology. Component-resolved diagnostics (45, 46) and prospective symptom recording via eDiary application were proposed as potential solutions to support the clinician in the detection of genuine sensitizations and confirmation of their clinical relevance (47). However, both tools generate rather large data sets which are difficult to work with in a busy clinical setting, providing an optimal opportunity for digitally facilitated and guideline-oriented decision support. The strength of this particular approach lies in a blended care approach combining the expertise and experience of the doctor in personal visits with digital monitoring support. Further, a looped design, facilitates a continuous re-evaluation based on real-time patient-recorded symptom data and information on the respective allergen exposure. The physician plays a strong role in this CDSS (named @IT-DSS) as it is customizable with regard to thresholds for test positivity according to personal experience and local environmental and epidemiological conditions (Figure 3). The @IT-DSS has been tested in a clinical pilot and multicenter study where the combination of face-to-face visits with symptom monitoring via eDiary showed promising results in terms of patient adherence, a common challenge for most monitoring apps. Although the daily use of the app for symptom and medication monitoring declined slowly over time, the observed drop in adherence was significantly lower than in other studies based on the spontaneous download of apps from the respective app stores (47). Further results, especially on the efficacy of the CDSS are expected to be published soon.

## Digital Decision Support for Asthma Care

A multitude of tools for asthma care have been developed, ranging from reminder systems (48), monitoring apps (49, 50), smart devices (51, 52) up to comprehensive digital care platforms (53). Although many of these technologies deliver valuable information for clinical decision-making, not all classify as a CDSS. Like for AR, also for asthma care there is a great heterogeneity in concepts, user groups and outcome assessments of digital health tools and CDSS (54). Interestingly, the impact of these systems seems to depend on the targeted user group. A systematic review on CDSS impact, when used by healthcare professionals with expertise in asthma management, reported a low effect on asthma control, mainly due to very low usage rates. The usability and user-centered design of CDSS are essential to achieve a sufficient adoption and high impact of the technologies (55). Similarly, a systematic review showed that CDSS are effective in improving care of patients with asthma and chronic obstructive pulmonary disease (COPD) when used in primary health care settings (56). The authors further underline that despite the positive impacts assessed in the randomized, controlled trials, the effects of the CDSS on user workload and efficiency, safety, costs of care, provider, and patient satisfaction remain understudied (56). Recently, the use of an electronic Asthma Management System (eAMS) improved the quality of asthma care for adult patients in a 2-year interrupted time-series study of usual care (year 1) vs. eAMS (year 2) at three Canadian primary care sites (34). However, further work on the identification of facilitators and barriers for uptake by clinicians is being done and randomized controlled trials assessing patients’ outcomes are still needed (34). Other promising concepts, such as the myAirCoach system (53), combine the use of a smart adapter for inhalers, with an indoor air-quality monitor, a physical activity tracker, a portable spirometer, a fraction exhaled nitric oxide device, and an app in one platform. Although these tools have only been tested in a small number of patients and not yet been included in a CDSS, they collect valuable information for personalized decision-making and it remains to be tested whether this concept can potentially be scaled up for a broader adoption. In addition, diagnostic approaches, such as serological multiplex tests for allergen- and virus-triggered asthma (57) could deliver important information for personalized asthma management, potentially supported by a CDSS.

## Digital Support Tools for Other Allergic Diseases

Several other tools have been developed to digitally augment allergy care namely supporting diagnosis and management in a primary care setting (58) or among junior clinicians (59), as well as for specific allergic diseases such as drug hypersensitivity (60–63), food allergies (64) and urticaria (65).

### Allergy Management Support for Primary Care

Based on literature review, focused interviews and testing in primary as well as secondary care patients, a group of allergists, dermatologists, GP and researchers from the Netherlands developed a guideline-based allergy management support system (AMSS) for allergy diagnosis and management in primary care settings (58). The AMSS interprets data from a 12-item multiple choice questionnaire with test results for allergen-specific IgE antibodies. After applying the algorithm to data from 118 patients, the authors identified 150 different diagnostic categories of AR, asthma, atopic dermatitis, anaphylaxis, food allergy, hymenoptera allergy, and other allergies. When comparing the AMSS outcomes with specialists’ recommendations as gold standard, an agreement of 69.2% (CI 67.2–71.2) was observed. In a clinical study on the implementation of the system, GPs showed a significant improvement in allergy diagnosis and reported a positive impact of the system on their clinical routine. However, the decision-making on medication and referral has not been affected by the use of the AMSS (66).

### Diagnostic Interpretation Support

With the aim of supporting the diagnostic decisions on AR among junior clinicians, a group of developers and researchers from India established a CDSS based on the clinical history and intradermal test results for 40 locally relevant allergens. The authors developed and validated the algorithm with data from 857 allergic patients and found that the CDSS differentiated the presence or absence of AR with an accuracy of 88.31% compared to the opinion of allergy experts (gold standard). Further, the study assessed the preferred CDSS model among junior clinicians who indicated to prefer a rule-based approach for its intelligible knowledge model (59).

### Drug Hypersensitivities

Several mobile applications have been created to assist doctors in the assessment of the causality, severity and preventability of adverse drug reactions (60). The use of a clinical decision support tool for non-allergists evaluating inpatients reporting penicillin allergy led to a twofold increase in penicillin or cephalosporin prescription compared to standard care (61). Similarly, a significant number of children could be de-labeled from penicillin allergy by primary care physicians following an algorithm for risk stratification and further work-up, including a telemedicine screening and single dose oral challenge (62). A systematic review has been conducted to assess the potential of computerized physician order entry systems with built-in clinical decision support for an improved management of drug hypersensitivity. The authors concluded, that the heterogeneity in recording of adverse events represents a considerable challenge for a unified interpretation of recorded data (63, 67). Further, an alert fatigue has been described in several studies due to a lack of alert specificity (67). As the alert systems are built to point out any potentially dangerous drug interaction of allergenic threat for patient safety, these alerts can become very numerous considering the large variety of drugs and potential interactions. Of course, clinical considerations of the individual patient need to be considered as well and studies showed that clinicians tend to ignore alerts in many cases. The frequent signaling of potential hazards may cause a certain fatigue among users which limits the possible impact of the system. Again, user-centered design seems to be essential, considering a balance between safety and alert frequency. This challenging task will need to be addressed in future developments and studies, recognizing particular needs, such as the support of primary care physicians in the management of chronic diseases where polypharmacy is the norm (68).

### Food Allergies

An initiative to support pediatricians in the work-up of patients reporting allergic symptoms related to foods has been created consolidating complex guidelines for the management of food allergies into five key steps. The development of the Food Allergy Support Tool further involved rapid-cycle improvement methods to create a CDSS facilitating food allergy management in a primary care setting. Interestingly a pilot evaluation showed that physicians were uncertain about the benefits of the system. The authors name the necessary active user initiation as a potential barrier for implementation (64).

## Improving Monitoring and Prevention by Connecting Stakeholders: Community Allergology

The strategy of making large data sets easily intelligible for specialized but also non-specialized healthcare providers, opens new opportunities for an efficient use of medical resources. In several countries, the numbers of specialized allergists are declining while there is a continuous increase of patients needing allergy care. This gap raises the pressure on primary health care workers and digital technologies represent a valuable tool for an intelligent distribution of work force and knowledge. This should ideally be supported by a digitally enabled availability of context-specific decision support, as guidelines may vary according to the clinical setting (primary care vs. specialist vs. tertiary care). By raising awareness at important community healthcare points such as pharmacies, an early identification of patients eligible for AIT can be fostered, always keeping in mind, that several significant barriers for the implementation of AIT still need to be overcome (69). Early interventions such as allergen avoidance can be efficiently implemented already at first contact with a primary health care physician and advice from specialists can be facilitated via remote consultations. But even beyond the purely medical field, a concept of community medicine can be revitalized via the use of comprehensive digital platforms. Patients can manage the access rights to their data and decide to share or donate them for research purposes or community projects, collaborating with environmental monitoring and public health institutions, a broad network of information is available for exploitation with the respective decision support tools.

Several of the above-mentioned support systems include the retrieval and storage of patient- and/or sensor-generated monitoring data. This provides the attending physician with comprehensive data sets for the evaluation of disease control during regular follow-up visits. Prospectively collected data on treatment adherence, quality of life, disease-specific symptoms and objective parameters such as sensor-recorded heart rate or sleep quality can be easily assessed in visual summaries or standardized scores (e.g., symptom and medication scores) which are generated in an automated fashion. At first sight, these digital platforms seem to clearly outperform traditional approaches. However, the impact of their use on diagnostic precision, treatment efficacy, safety, quality of life and treatment costs needs to be objectivized and studied in more detail with regard to allergic diseases as it has been already done in other specialties and areas of medical care (70–73).

## Perspectives

In summary, digital technologies offer a vast potential to support clinicians in their actions for prevention, diagnosis, treatment, and monitoring of allergic diseases. Many different concepts are under development and in different validation stages, which opens a promising perspective for the next years. However, no tools are currently commercially available yet and time-consuming evaluations are necessary to enable the registration as a medical product. As CDSS may have a significant impact on key decisions for patient care, they need to be rigorously tested for applicability and usability in order to support clinicians in making the best choices for their patients. In addition, the interoperability with existing software systems and a smooth integration in clinical routine are significant challenges for a successful implementation. More real-life experiences and clinical studies will need to be conducted in order to extend our knowledge and foster a solid adoption in of digital support tools in the clinical routine.

## Author Contributions

SD supervised and performed the literature research and wrote the major parts of the manuscript. EP and MM contributed to literature screening, writing of the manuscript and formatting. PM carefully reviewed and corrected the manuscript. All authors contributed to the article and approved the submitted version.

## Conflict of Interest

The authors declare that the research was conducted in the absence of any commercial or financial relationships that could be constructed as a potential conflict of interest. The reviewer DR declared a past co-authorship with the authors, SD and PM, to the handling editor.

## Abbreviations

AH: antihistamines
AIT: allergen-specific immunotherapy
AMA: American medical association
AMSS: allergy management support system
AR: allergic rhinitis
ARIA: allergic rhinitis and its impact on asthma
CDSS: clinical decision support system
COPD: chronic obstructive pulmonary disease
DSS: decision support system
eAMS: electronic Asthma Management System
EHR: electronic health record
GP: general practitioner
HCP: health care professionals
INCS: intranasal corticosteroids
PDSS: pharmacy decision support system
VAS: Visual Analogue Scale.
